# Student midwives’ experiences of Ubuntu principles in midwifery care at Vhembe District

**DOI:** 10.4102/hsag.v30i0.2924

**Published:** 2025-06-10

**Authors:** Nthuseni T. Munzhedzi, Khathutshelo G. Netshisaulu, Dorah U. Ramathuba

**Affiliations:** 1 Department of Advanced Nursing Science, Faculty of Health Sciences, University of Venda, Thohoyandou, South Africa

**Keywords:** application, Ubuntu, principles, provision, midwifery, services, student midwives, experiences

## Abstract

**Background:**

Ubuntu, a philosophy of human dignity, moral high ground, compassion, interconnectedness and community, significantly enhances ethical and caring practices in nursing and midwifery.

**Aim:**

This study explored student midwives’ experiences in applying Ubuntu principles in midwifery services in Vhembe District, Limpopo province.

**Setting:**

The study was conducted at the Vhembe District in Limpopo province, focusing on Level 4 student midwives learning at two tertiary nursing education institutions.

**Methods:**

This study used a qualitative, exploratory and descriptive approach. The study was conducted in three selected hospitals of Vhembe District, which were purposively selected. Thirty Level 4 student midwives from two tertiary nursing education institutions were purposefully selected from three district hospitals. Data were collected through face-to-face, semi-structured interviews, and thematically analysed using Tesch’s eight-step approach. Credibility, transferability, dependability and confirmability ensured trustworthiness. Ethical principles were rigorously followed throughout the research process.

**Results:**

The findings revealed both positive and negative experiences. Student midwives reported patient support, mutual respect and collegiality, alongside discrimination and abuse. These contrasting accounts point to a disconnect between Ubuntu principles and actual clinical practice, highlighting the gap between ethical ideals and the lived realities of midwifery care.

**Conclusion:**

While some areas of clinical practice support Ubuntu values, there are significant gaps. Student midwives are exposed to positive and negative experiences in the practice of Ubuntu in midwifery services. The study recommends Ubuntu principles in midwifery practice and education to promote human-oriented and ethical care.

**Contribution:**

The study advocates for the consistent affirmation of Ubuntu in midwifery practice and education to promote ethical integrity and enhance the quality of maternal care.

## Introduction

According to van Asamoah and Yeboah-Assiamah (2019), Ubuntu is an African humanist and philosophically motivated viewpoint on the human situation and how people behave towards one another. The Ubuntu principle helps nurses and midwives understand that another person’s humanity is a part of their own (Kauka 2018). As Ubuntu is determined by the degree of a person’s relationships with or behaviour towards other people, this interpretation or viewpoint of the philosophy is seen as guaranteeing that nurses and midwives treat patients with the respect to which all human beings are entitled (Ewuoso & Hall 2019, Van Breda, 2019).

*Ubuntu* also includes actions that are clearly beneficial to the community, whether they are as basic as lending a hand to a stranger in need or more intricate ways involving other people (Muhammad-Lawal et al. 2022). In addition, during their training, student midwives will be exposed to the Ubuntu ideology, which emphasises humaneness, respect and dignity in patient care (Muhammad-Lawal et al. 2022). Furthermore, meeting the needs and expectations of their communities by delivering nursing care and midwifery services while taking into account the cultural values and beliefs of the communities is a requirement of both professional and ethical standards for nurses and midwives. Prenatal care, intrapartum care and postnatal care are examples of midwifery services (Twumasiwaa-Boateng & Yakong 2022). Nursing and Ubuntu are closely related. They both highlight that care is the most important aspect of the nursing profession. Ubuntu sees people based on their ability to inspire others. Ubuntu emphasises community, with the motto ‘I am because I belong’. A nurse can be raised in and born into an Ubuntu community, where they can develop the values of compassion, dedication and belonging – all predicated on the communitarian philosophy of Ubuntu. This is comparable to how someone can be born into a community that is centred around Ubuntu. According to a philosophical framework proposed by the Ubuntu principles and values, senior and junior nurses can work together in the community (Memela 2021).

In addition, the Ubuntu philosophy would familiarise student midwives with the expected or required humaneness, respect and dignity in their treatment of patients during their training (Muhammad-Lawal et al. 2022). Furthermore, it is both professionally and ethically required that nurses and midwives should meet their communities’ expectations and needs by providing nursing care and midwifery services with due consideration to the communities’ cultural values and beliefs. Such midwifery services include antenatal care, intrapartum care and postnatal care (Muhammad-Lawal et al. 2022).

### Aim of the study

The aim of this study was to determine the experiences of student midwives regarding the application of Ubuntu principles during provision of midwifery services in Vhembe District, Limpopo province.

## Research methods and design

### Study design

The processes, procedures, strategies and research instrumentation that the researcher prefers to use in order to solve the research problem and accomplish the study’s overall goals are all part of the research design (Bryman 2021). To improve its research methodology, the study also used an exploratory and descriptive research design to better understand the phenomenon of Ubuntu and the experiences of student midwives in the Vhembe District Municipality with regard to applying Ubuntu principles while providing midwifery services.

While there are some similarities between exploratory and descriptive research designs, there are also some differences (Corbin & Strauss 2019). Descriptive research offers more specific information about the relational variables of a situation or social settings and further focusses on answering the why and how questions, whereas exploratory research primarily presents a holistic picture regarding the details of a situation, even though the two approaches may be combined in practice (Anderson 2019). In order to learn more pertinent details about the experiences of student midwives in relation to the application of Ubuntu principles during their midwifery services in the Vhembe District Municipality, the researcher used both exploratory and descriptive research design approaches in this study.

### Setting

A study setting is defined by Grove, Grey and Burns (2018) as the natural, somewhat controlled or highly controlled physical location where the research was carried out. The researcher conducted the investigation at the University of Venda (Department of Advanced Nursing Science) and Thohoyandou Nursing Campus which are in Thulamela Local Municipality (LM) in the Limpopo province’s Vhembe District.

From the seven hospitals in Vhembe District, only three were purposively selected, namely, Tshilidzini, Donald Fraser and Siloam hospitals. The rationale for purposively choosing these three hospitals is that midwifery nursing students from both selected training institutions (i.e. Thohoyandou Nursing Campus and the Department of Advanced Nursing Science, University of Venda) are placed in these selected hospitals for midwifery clinical learning.

### Study population and sampling strategy

A population of the study relates to the whole group of persons, units, events or objects that are bound by common characteristics and qualities that are of relevance and interest to the study in terms of meeting the criteria that the researcher has determined before undertaking the study (Leedy & Ormrod 2020). The accessible population in this study are a segment of the entire research population and consist of all midwifery students at both the Thohoyandou Nursing Campus and the Department of Advanced Nursing Science, University of Venda. Therefore, the targeted population of the study were only the Level 4 midwifery students from both the Thohoyandou Nursing Campus and the Department of Advanced Nursing Science, University of Venda who were placed at the three district hospitals (i.e. Tshilidzini Regional Hospital, Donald Fraser District Hospital and Siloam District Hospital) for their clinical learning.

Sampling is described as the process by which the researcher selects a group of the most representative participants from an identifiable population to obtain information regarding a problematic phenomenon based on some predetermined criteria (Ary et al. 2019). In this study, the representative group of participants were chosen according to the non-probability purposive sampling method. This technique relies on the researcher’s own judgement as the sole determinant of *how* and *why* a certain type of participants is deemed relevant for involvement in the study (Saunders, Lewis & Thornhill 2019). Therefore, the researcher opted for the purposive sampling strategy because of her ample knowledge and understanding of the research environment in the Vhembe District. The purposive sampling option was also influenced by the fact that it does not guarantee any chance for participation in the study, which is advantageous for elimination of possible researcher bias (Polit & Beck 2021).

### Inclusion criteria

The following inclusion criteria were applied for inclusion of prospective midwifery students in this study:

Fourth-level midwifery students from both the Thohoyandou Nursing Campus and the Department of Advanced Nursing Science, University of Venda who were placed at the three district hospitals, and willing to take part in the study.

### Exclusion criteria

Meanwhile, the following criteria or range of considerations rendered any prospective participants excluded from any involvement in the research study:

First-, second-, and third-year midwifery students from both the Thohoyandou Nursing Campus and the Department of Advanced Nursing Science, University of Venda.

The sample size comprised of 30 participants. Of these, 10 were midwifery students from the Thohoyandou Nursing Campus and the Department of Advanced Nursing Science, University of Venda, while the other 20 were from the Thohoyandou Nursing Campus.

The researcher verified with the participants if they understood the purpose of the research before collecting data. The risks, benefits and participants’ rights were discussed at length before they consented to participate in the study. Researcher explained the goal of the study to participants and obtained their completed informed permission forms before the interview started. All participants were thus given the chance to agree or disagree to take part in the research study (Mntonintshi-Mketo, Netangaheni & Lefoka 2024).

### Data collection methods and procedures

The researcher developed and used an interview guide, which comprised of a logically organised list of questions. The interview guide questions were based on the objectives of the study.

### Data collection

Both the information sheet and informed consent form provided relevant information, including a full disclosure concerning the study purpose and the use of its findings (Hennink, Hutter & Bailey 2020). Seeking permission was also a means to build trust and rapport between the researcher and her participants (Samuel & Richie 2022). The individual semi-structured interviews were held at pre-arranged venues at each of the sampled healthcare facilities. These venues were in the secluded sections of the hospitals to avoid a disruption of the daily operations of these hospitals. At the beginning of each session, the researcher introduced herself and immediately made a disclosure of the study. The researcher requested the participants’ permission to audio-record the interviews, which enabled the researcher to ensure that no participant-related information or responses were omitted or missed. A tape recorder was used by the researcher to make sure that she did not omit any information given by the interviewee. Probing questions were used during the interviews to enable participants’ more detailed responses concerning the subject matter at hand. At the end of each interview session, the researcher thanked the participants for their time and their positive involvement and contributions in the study. All transcriptions (audio records, notepad notes, copies of consent forms) were anonymised.

### Data analysis

Data analysis is the systematic process of organising, categorising and reducing collected data to translate or convert such information into usable text and images for developing a framework of the findings (Botma et al. 2022). Data were analysed using Tech’s eight steps in the coding process as recommended by Creswell (2020) and in conjunction with the thematic analysis method. The eight steps are discussed as follows:

#### Step 1: Organising and preparing the data analysis

Creswell (2020) advises on transcribing all recorded or captured data verbatim to improve the analysis process. The researcher transcribed all the audio-recorded interviews verbatim in Microsoft Office to ensure safe storage and readability.

#### Step 2: Reading or looking at all data

The researcher carefully read all the verbatim transcripts to attain a comprehension of data segments and their meaning. The notebook was used to record the meaning and understanding that surfaced throughout the reading of the verbatim transcripts, along with the related thoughts. By carefully and repeatedly reading the participants’ transcripts over an uninterrupted period, the researcher processed and understood data in its totality to ensure quality data analysis and record all notes and thoughts in real time as they came to mind.

#### Step 3: Coding all the data

Based on the existence or frequency of concepts in the verbatim transcripts, the researchers then scaled down data for coding while listing all topics that emerge. In the process, grouping similar topics and separately clustering those that are dissimilar. The researcher recorded notes and thoughts of the collected data in the margins of the articles where the verbatim transcripts appear.

#### Step 4: Generating description and all themes

The researcher once again analysed the transcriptions with concerted focus on the codes, which existed from the frequency of the concepts (mental picture codes when reading through) Questions such as ‘*Which words describe it*?’, ‘*What is all about*?’ and ‘*What is the underlying meaning*?’ will guide the researcher in this step.

#### Step 5: Representing the description and themes

The researcher abbreviated the topics that emerge as codes, writing the abbreviation next to the appropriate segments of the transcription. Thereafter, the researcher differentiated the codes by including meaningful existences of specific coded data. The analysis process encapsulated recording these codes in the margins of the notebook using a different-coloured pen from that used in Step 3, thus enhancing visual differentiation and supporting the systematic organization of themes.

#### Step 6: Developing the themes and subthemes

Themes and subthemes were developed from coded data and the associated texts. And the complete list was reduced by grouping topics that are related to each other in order to create meaning of the themes and subthemes. To understand the meaning of the themes and subthemes, Kohlberg’s Theory of Moral Development was applied. This entailed aligning the meaning of each of the three phases to define a particular theme or sub-theme. For example, the preconventional phase is when moral development is taking place, and moral actions of student nurses are characterised by fear of punishment (stage 1) or personal benefit (stage 2).

#### Step 7: Comparing the codes, topics and themes for duplication

In this step, the researcher reviewed the work from the beginning, checking for duplication and refining codes, topics and themes where necessary.

#### Step 8: Recording the existing data, where necessary

The data associated with each theme were assembled in a single column to complete the preliminary analysis. Liaison between the researcher and an independent coder confirmed the themes and subthemes of the researcher prior to the researcher producing the final research.

## Measures to ensure trustworthiness

### Credibility

Credibility relates to the confidence of the researcher in the truth value of the data generated and the intensity according to which the self-same data were interpreted (Botma et al. 2022). Credibility also involves the extent of the study results’ believability or acceptability of the study results from the viewpoint of the research participants. In addition, credibility (known as validity in quantitative studies) is ascertained by the participants’ authentication, validation, corroboration and approval of the findings. The higher the outcome of these and the higher the study’s validity. The researcher ensured credibility by member checking, building rapport with the participants and literature control.

Credibility was established by building rapport and trust with the participants. First and foremost, the researcher did not just begin by asking questions in the interview. Rather, she warmly introduced herself at the beginning of each session and fully disclosed the study and its purpose. Through prolonged engagements, the researcher further spent more time with the participants outside of the formal interview sessions to understand their worldview and rationale for their experiences and responses to the interview questions (Johnson, Adkins & Chauvin 2020).

In addition, member checking (which could be viewed as complementary to building rapport with the participants) was applied with the researcher’s post-interview follow-up for further clarity on participants’ answers to certain questions, which the researcher might have missed. Member checking was employed to validate the obtained data through discussions with the participants, who were allowed to react to their responses when analysed data were referred to them for their further review, validation or correcting the generated themes and categories where it was necessary (Kumar 2019). The researcher ensured credibility by consistently pursuing various possible interpretations of the analysed data.

### Transferability

Transferability is premised on the findings’ applicability or generalisability to other participants or contexts facing similar challenges as in the original study (Bryman 2021). In this study, the results are expected to produce high generalisability and transferability to other contexts or settings because of the homogeneous groups of student midwives represented in the study. Although transferability of research findings is not always a possibility in qualitative research studies (Leedy & Ormrod 2020), the researcher has kept records of the research processes, which will enable interested researchers to fully understand the study and its current approaches for possible application or replication in their own respective settings.

### Conformability

Confirmability relates to the degree of the study results’ corroboration by independent professionals or experts in the field of inquiry (D’Angelo et al. 2019). Confirmability also relates to the extent of data congruity, relevance and accuracy and clarity of meaning (Leedy & Ormrod 2020). The data should reflect the participants’ voice, and not the perceptions, biases or preferences of the researcher (Saunders et al. 2019). Therefore, the researcher consulted a professional research methodology practitioner and coder for independent verification of the relevance and accuracy of the findings, as well as confirming the agreeability of the findings and conclusions reached. Confirmability was further ensured by capturing all interviews on audio to retain the original state of the participants’ statements before their analysis and interpretation. The researcher used the follow-up or probing questions to preclude any assumptions concerning the participants’ responses.

### Dependability

Dependability is premised on the view of the study’s evidence becoming repeated, irrespective of context, circumstances and conditions external to the initial research study (Anderson 2019). Dependability was applied in this study by asking the same questions to all participants, irrespective of the time and circumstances of each interview session (Ary et al. 2019). Furthermore, the researcher used the probing strategy in a way that did not change the original meanings and contexts of the questions.

### Ethical considerations

This study was guided by social science ethical consideration and measures to ensure trustworthiness. Furthermore, ethical clearance was granted by the University of Venda’s Human and Clinical Trials Research Ethics Committee (HCTREC), (reference no: FHS/22/PDC/10/1206) which provided the required authorisation for the data collection process to commence. Thereafter, permission was granted by the Limpopo Department of Health, Vhembe District Department of Health managers and the Department of Health District Managers of the three research sites. Once permission was granted by the hospital authorities, appointments with the three hospitals were made, with a schedule of possible dates for the semi-structured interviews. This enabled the researcher to avoid interrupting student midwives’ daily work schedules at the hospitals. To protect participants’ identity and information, the researcher created a file using her computer, which is protected by a password. The researcher will keep the data for a period of 5 years. Thereafter, it will be permanently erased.

## Results

The demographic data of the participants, indicating their age, gender, level of training, and hospital placement, are depicted in Table 2. To maintain confidentiality and anonymity, the participants were assigned codes from P1 to P30. Of the 30 participants, 3 were younger than 22 years; 19 were between the ages of 22 and 23 years, and 8 were aged 24 years or older. Among the total participants, 25 were female and 5 were male, reflecting a strong female representation in the cohort. All participants were at Level 4 of their training, indicating a homogenous academic standing. In terms of clinical placement, 14 participants were based at Hospital B, 9 at Hospital A, and 7 at Hospital C. The data collection took place in environments familiar to the participants, and while language preferences were not specified, all participants shared a similar training context. Participants were identified only by pseudonyms to ensure anonymity, and their demographic characteristics provide a valuable context for interpreting their contributions in the study.

**TABLE 1:** Participants’ demographic characteristics.

Pseudonym	Age (years)	Gender	Level of training	Hospital
Participant 1	22	Male	4	HB
Participant 2	23	Female	4	HB
Participant 3	23	Female	4	HB
Participant 4	23	Female	4	HA
Participant 5	22	Female	4	HA
Participant 6	25	Female	4	HA
Participant 7	23	Female	4	HA
Participant 8	22	Female	4	HA
Participant 9	30	Female	4	HA
Participant 10	25	Female	4	HA
Participant 11	22	Female	4	HA
Participant 12	24	Female	4	HA
Participant 13	30	Female	4	HA
Participant 14	23	Female	4	HB
Participant 15	22	Female	4	HB
Participant 16	21	Female	4	HB
Participant 17	26	Female	4	HB
Participant 18	22	Female	4	HB
Participant 19	22	Female	4	HB
Participant 20	22	Female	4	HB
Participant 21	23	Female	4	HB
Participant 22	24	Male	4	HB
Participant 23	21	Female	4	HB
Participant 24	22	Female	4	HB
Participant 25	22	Female	4	HC
Participant 26	21	Female	4	HC
Participant 27	25	Male	4	HC
Participant 28	24	Male	4	HC
Participant 29	25	Male	4	HC
Participant 30	23	Female	4	HB

Note: Data were collected from a sample of 30 participants.

HA, Hospital A; HB, Hospital B; HC, Hospital C.

**TABLE 2:** Emerging themes and sub-themes from participants’ experience of Ubuntu principles.

Themes	Sub-themes
1. Positive experiences of Ubuntu application	1.1 Patient support 1.2 Teamwork and collegiality 1.3 Respect of patients 1.4 Non-discrimination
	2. Negative feedback on Ubuntu	2.1 Shouting of patients by senior midwives 2.2 Fair versus unfair treatment based on race and nationality 2.3 Disrespect of student midwives by senior midwives 2.4 Psychological and emotional abuse of patients 2.5 Physical abuse of patients 2.6 Shortage of staff 2.7 Poor infrastructure

### Theme 1: Positive experiences of Ubuntu application

Positive experiences of Ubuntu can be related to acts of goodwill, ordinary good manners and treating patients as unique being with respect and dignity without prejudice and discrimination. Participants related their positive experiences about application of Ubuntu principles based on patient support, teamwork and collegiality, respect of patients and non-discrimination. The development of this theme was framed by the conventional phase of Kohlberg’s Moral development theory. At this stage, moral reasoning is shaped by the society’s expectations of good behaviour. These expectations include supporting patients, working in a team and providing respectful, non-discriminatory care.

#### Sub-theme 1.1: Patient support

The participants in the study revealed that both student midwives and few senior midwives are able to display Ubuntu principles during provision of midwifery services. Participants revealed that they are supportive and caring and also provided patients with appropriate information, which enabled them to understand their health conditions and responded positively as expected. This is supported by the following statement:

‘Most of student midwives display good nurse-patient relationship and patients support is always good, does not shout at patients instead, patient is given full explanation before and starting the delivery procedure and also explain the expectation thereof to gain cooperation.’ (Participant 17)

While among the senior midwives, the findings revealed that only a few of them supported patients, hence the application of Ubuntu. This is supported by the following narrative statements:

#### Sub-theme 1.2: Teamwork and collegiality

Participants in the study reported teamwork as one of the aspects they experienced, which positively impacted the provision of midwifery services to patients. Thus, midwives and student midwives worked harmoniously with each other, resulting in quality care provision to patients despite the shortage of staff. The following were echoed by the participants:

‘During antenatal, I experienced the importance of team spirit while allocated in the antenatal ward which was very full and short staffed and there we work very well and stick together as a team.’ (Participant 3)

‘I was suturing the episiotomy to the patient who was not co-operating and called for help from a senior midwife who came and supported the patient by emphasising the importance of cooperating to ensure that suturing of the perineum is done well.’ (Participant 28)

#### Sub-theme 1.3: Respect of patients

Participants highlighted that respect is one of the values of Ubuntu as described in the context of respecting patients during care provision. In this regard, student midwives shared their experiences of respect between the midwives and patients. The respect from the senior midwives was given to ‘high profile patients’, while another participant noticed that the respect was provided in the antenatal unit. Noteworthy student midwives who described the positive experience of respectful maternity care were from the same institution.

The quotes to support are as follows:

‘Respect is only given as per prejudice and high profile cases are treated with respect.’ (Participant 20)

‘During antenatal care senior midwives treat patients with respect and dignity. Labour ward where high risk patient is booked for elective C/S either due to obstetric and medical conditions, but the senior midwives ensure that everything of the patient went well.’ (Participant 15)

#### Sub-theme 1.4: Non-discrimination

According to Respectful Maternity Care, all patients should be treated as unique beings, with respect and dignity, irrespective of their culture, ethnicity and religion without discrimination or prejudice. Therefore, non-discrimination was reported by the participants, who revealed that there are senior midwives who still displayed Ubuntu during provision of healthcare services. They highlighted that patients are treated equally, irrespective of their culture and religion. Their quotes to support this sub-theme are shown below:

‘They are those senior midwives who are still treating patients equally irrespective of their culture and believe.’ (Participant 23)

‘Good quality care is provided in maternity ward and patients are well taken care of with fair treatment to all, irrespective of their culture and beliefs.’ (Participant 29)

### Theme 2: Negative feedback on Ubuntu

The second theme aligned with the major theme of Ubuntu principles during the provision of midwifery services was the negative feedback on Ubuntu. The development of this theme was framed by the first phase of Kohlberg’s Moral Development stage. At this stage, the student midwives observed negative feedback on Ubuntu principles because of the consequences of opposing senior midwives and/or self-interest.

In this regard, the participants expressed their views on negative feedback regarding Ubuntu principles. The negative feedback on Ubuntu subsumed five subthemes, illustrating the lack of Ubuntu among the senior midwives. Patients were shouted at by senior midwives, fair versus unfair treatment based on race, disrespect of student midwives, physical abuse of patients, and psychological and emotional abuse of patients by senior midwives.

#### Sub-theme 2.1: Shouting at patients by senior midwives

In this study participants revealed the opposite; instead, during provision of care, senior midwives shouted at patients by saying hurtful things unnecessarily. Usually, it led to negative outcomes. This was evidenced by quotes from participants who found the constant shouting of patients and calling them using bed numbers or diagnoses that did not embody Ubuntu. The excerpts supporting the subtheme are shown next.

The finding described how nurses would shout at patients:

‘Ubuntu principles are not applied. Nurses are shouting to the patients’ telling patients that “this is not your home!”. They are also rude to the patients.’ (Participant 2)

‘Nurses shout at patients instead of speaking with them nicely and they also call patient by bed instead of their real names.’ (Participant 3)

‘Patients are not given opportunity to express themselves.’ (Participant 7)

#### Sub-theme 2.2: Fair versus unfair treatment based on race and nationality

The participants reported unfairness regarding care provision to South Africans and Zimbabweans, whereas Indians received special care versus South Africans.

This is shown in the following quotes:

‘People are different, and some abide to Ubuntu principles, discriminations of patients for example Zimbabwean patients are not attended in time, just sit and wait for a long period, to access medical treatment they are shouted at, and the approach is bad as compared to other cultures.’ (Participant 9)

‘There is discrimination in labour ward of the hospital, Indians and South African are not treated equally, and the Zimbabwean is insulted in front of other patients.’ (Participant 24)

#### Sub-theme 2.3: Disrespect of student midwives by senior midwives

Participants reported that the respect of student midwives is lacking in maternity wards. Senior midwives’ yelling at student midwives in front of patients or other professionals is an example of how they are disrespected and not treated like special beings. This type of attitude led to a negative impact on the student’s midwives, who develop low esteem and low self-confidence in their daily activities. This subtheme resonated with phase 1 of Kohlberg’s Moral development theory, stage 1 of the preconventional phase, where the student could not act on their moral dilemma because of fear of consequence of being labelled or not getting signatures. The issue of disrespect and failure by the student midwife to act on a moral dilemma is described by the participants whose quotes are as follows:

‘The student was trying to message the patient who was in labour and was shouted at by the senior midwife who said “Do you want to spoil our patients!” [*U do ri tshinyela vhalwadze hoyu*] “are you going to message all these patients in labour?” and I just kept quiet for that and comply with the ward rules to avoid being labelled and not get signatures after completion.’ (P6, 25, Female, from HC)

‘I was shouted at when we need help like “matshudenihaya a bora!” [*these students are boring*] only the students from university students are highly considered and get more privileged to be taught versus the college student, I felt embarrassed and demotivated.’ (Participant 9)

#### Sub-theme 2.4: Psychological and emotional abuse of patients

Participants described how patients were psychologically and emotionally abused through verbal threats. They were told that maternal services would not be provided to them. They were shouted at, humiliated, and embarrassed irrespective of the stage of labour and severity of pain. These findings revealed a lack of Ubuntu in the maternity ward.

The statements of the student midwives illustrate how maternal services may fail to address patient pain:

‘“Due to different pain tolerance, some patient may be screaming even though is still latent phase 2cm dilated jumping all over and crying for help, Instead, nurses will shout to the patient that this is how to give birth and you must tolerate instead of giving patient analgesic to relieve pain.’ (Participant 21)

‘“The student was suturing episiotomy to the patient who was not cooperating and call for help from the senior midwives who came and told the patient her that” “You will leave with your perineum and will see if your husband will be happy to see you like this”, after this the patient was cooperative and suturing was completed after change of behaviour.’ (Participant 28)

#### Sub-theme 2.5: Physical abuse of patients

Participants reported physical abuse of patients by senior midwives in labour ward, claiming that the intention is to save the life of the unborn baby. This was usually done to the primigravida patients who were not well informed on what to expect in labour. These were supported by the following statements:

‘“I was in labour ward while one of the patients who was giving birth, requested to push the baby out and not complying, instead the senior nurse forcefully open the legs of the patient by pulling the legs away to open the legs and also shouted to the patient”. until the baby delivered alive.’ (Participant 22)

Another participant similarly noted that such practices were very common:

‘During labour patients are opened and stretched legs forcefully with support from different nurses especially to the primigravida and non-cooperating.’ (Participant 27)

#### Sub-theme 2.6: Shortage of staff

The participants expressed their views regarding the importance of the availability of human resources to ensure quality maternal services. In their descriptions they revealed that they are working in an environment that is not conducive because of the shortage of staff. They are providing midwifery services without supervision because of the staff shortage and ‘which they view as trial-and-error learning’ to safe both the life of the mother and the baby. As a result, they emphasised the significance of having enough qualified, experienced and skilled midwives to guarantee high-quality midwifery services. This will lead to a reduction of burnout and shouting at patients and a result of exhaustion that violated Ubuntu principles.

This is supported by the following narrative statements:

‘All shortage of staff needs to be corrected … Every maternity ward should have enough staff to ensure provision of quality care and application of Ubuntu principle.’ (Participant 12)

‘Shortage of staff during admission and preparation for caesarean section where everything was done without supervision which is a violation patients’ rights that jeopardise Ubuntu application.’ (Participant 3)

#### Sub-theme 2.7: Poor infrastructure

Participants revealed that they are working in an environment that is not conducive because of poor infrastructure. Curtains are used to divide the beds. They also revealed the issue of overcrowding that resulted in a lack of confidentiality, dignity and privacy of patients. As a result, these hinder the quality provision of maternal services and Ubuntu principles. These findings are supported by the following narrative statements:

‘Bad infrastructure in labour ward which is too small that always lead to overcrowding with patient, no privacy as the bed is divided by curtains due to lack of space.’ (Participant 1)

The finding also showed that adequate infrastructure ensured dignified quality care from another hospital.

## Discussion

### Theme 1: Positive experiences of Ubuntu application

According to the study’s findings, there have been some beneficial experiences with using Ubuntu when providing midwifery services. Patients’ support during these experiences resulted in higher-quality care because midwives massaged labouring women, which induced contractions and made delivery easier. This is consistent with the findings of Maputle (2018), who found that midwives’ assistance to patients minimises problems and lowers the need for medical treatments. The results showed that a pleasant Ubuntu application experience embodies the concept of togetherness, which includes moral support and teamwork (Dehghani, Mosalanejad & Dehghan-Nayeri 2019). The unity that characterises Ubuntu was seen by several of the senior midwives in their support of the patients. Similar views were expressed by Maputle (2018), who underlined that senior midwives’ provision of patient support during labour is crucial because it shortens labour and demonstrates the use of Ubuntu while lowering the need for medical procedures such as caesarean sections and their complications.

Vaajoki et al. (2023) assert that delivering safe, efficient and women-centred maternity care requires teamwork. As a result, the study also discovered that collaboration and camaraderie guaranteed the student midwives a satisfying experience when using Ubuntu. According to additional research conducted in South Africa by Tshosane (2018), who also employed an exploratory qualitative study design, this kind of collaboration between senior midwives and student nurses is what Ubuntu should represent.

Senior midwives and student midwives demonstrated team nursing while providing maternity services. Effective teamwork created a healthy work atmosphere and enhanced patient safety, all of which were reflected in the successful application of Ubuntu values. Özden et al. (2019) provided evidence for this, stating that team spirit is defined as the daily operations of maternal healthcare impacting the workplace culture to guarantee high-quality midwifery services. This favourable experience is further supported by the research conducted by Tshosane (2018), who discovered that collaboration between student nurses and senior midwives or other nursing team members facilitated the use of Ubuntu.

Along with helping the student midwives, the senior midwives’ respect for the patients in the labour ward and during prenatal care was another example of how Ubuntu values were positively used. Other research disputes this conclusion, even if Maputle (2018) describes the ideal application of Ubuntu as characterised by this demonstration of patient respect. For instance, research by Jafari et al. (2019) and Zali (2020) revealed that midwifery care is defined by patient contempt. Furthermore, mentees such as student midwives imitate this disregard for patients, which leads to a persistent culture of disrespect in the midwifery field, according to Jafari et al. (2019).

Given the discrepancy between the results of this study and those of studies by Jafari et al. (2019) and Zali (2020), more research is necessary to get definitive proof about the use of respectful maternal care. Every patient has the right to obtain medical care without facing discrimination, as stated in the Patient Rights Charter of 2023 (Papinaho, Häggman-Laitila & Kangasniemi 2022). The findings also demonstrated that because patients’ cultural inclinations differed and they weren’t all treated equally, they experienced prejudice. Some research in other contexts dispute this finding, even though it demonstrates the provisions of the Patients’ Rights Charter, the Maternity Guidelines in South Africa of 2023 (Papinaho et al. 2022) and the Batho Pele Principles, which state that all patients should be treated without discrimination.

According to a study performed in the United States, minority women are more likely to encounter unequal maternal care delivery (Adebayo et al. 2022). The study also discovered that student midwives did encounter discriminatory care services, indicating unfavourable experiences with Ubuntu, even while non-discriminatory maternal services were provided. Ubuntu, which includes the equality principle, is not exemplified by the unfavourable experience the student midwives recounted as a result of unequal treatment (Dehghani et al. 2019). The discovery of both fair and unjust treatment based on race and nationality is not exclusive to the South African environment, even in the absence of Ubuntu.

A study conducted in the United States by Adebayo et al. (2022) confirmed the same findings, indicating that minority African American women experienced prejudice because of their lack of health insurance, socioeconomic status and colour. Consequently, unequal and discriminatory treatment increased the likelihood of maternal problems for black women (Adebayo et al. 2022).

### Theme 2: Negative feedback on Ubuntu application

Participants also discussed negative experiences they had when applying the Ubuntu principles. Senior midwives shouting at patients, treating patients differently based on their race and nationality, treating student midwives disrespectfully, physically and psychologically abusing patients, understaffing, and having subpar facilities were all contributing factors to these negative experiences. The results of this study also showed that student midwives looked up to the professional nurses as role models because of the senior midwives’ yelling at patients. However, some of the professional midwives’ unfavourable behaviours had a detrimental effect on their experiences and optimistic expectations in the application of Ubuntu principles. These practices included yelling at patients in an impolite and unprofessional way, which the student midwives did not think exemplified Ubuntu.

In contrast, a study conducted by Mulaudzi et al. (2022) found that junior nurses, including student midwives, implement Ubuntu differently than older nurses who may have access to the concepts earlier in their employment. In contrast to the more experienced professional midwives, it is implied that the younger nurses were more likely to yell at patients. However, Kosicki, Tomberg and Bradley (2018) pointed out that younger student midwives’ failure to adopt Ubuntu principles was mostly caused by a lack of suitable leadership direction, role modelling and mentorship ability, rather than their age, which contrasts with the findings of Mulaudzi et al. (2022).

Unfortunately, the contempt, condescension and abuse of maternal patients by professional midwives is reflected in these shortcomings and a lack of leadership (Naidoo & Ramphal 2019). According to the participants, patients are treated both fairly and unfairly based on their nationality and race. In describing how they understood and applied the Ubuntu principles, the participants pointed out that they were frequently broken, as evidenced by the discrimination against South Africans and Zimbabwean nationals seeking medical care, as well as their Indian counterparts who received some preferential treatment.

Such discriminatory actions are against the law and unethical, which violates the Patients’ Rights Charter and the Batho Pele principles (Department of Public Service & Administration [DPSA] 1997), as well as the constitutionally guaranteed Right to Equal Treatment (Department of Health 2018). The findings that discriminated against Zimbabwean nationals by making them wait longer to receive maternity healthcare services are particularly noteworthy. The negative portrayal of midwifery and the nursing profession in general in the media is supported by such xenophobic, racist and ethnic behaviours (Sobuwa 2022; Sompane 2022).

Despite serving as an example of the absence of Ubuntu implementation, the physical abuse finding is consistent with research from Nigeria, Ghana, Guinea and Myanmar, where at least 30% of women experience verbal and physical violence both before and after giving birth (Sen et al. 2018). Furthermore, all patients from low socioeconomic and educational backgrounds received disrespectful treatment. In a similar vein, Sen et al. (2018) found that women who were primarily young, single, and had less education were more likely to experience abuse in Guinea, Ghana, Nigeria and Myanmar. The prejudice against poorer patients, who were also shown to be mistreated in a manner similar to that described in the study conducted by Sen et al. (2018), was linked to these findings of discrimination against younger women. Regardless of the presumed power dynamics and relations, the latter is obviously a superb example of condescension, which goes against the idea of treating others with humanity and equality (Schryen et al. 2020).

The results showed that while Zimbabwean and young patients were not treated with respect, certain patients – such as those who are ‘high profile’ and ‘Indian’ – were. This condition aligns with the results of a study conducted in a province in South Africa by Malatji and Madiba (2020), which found that women admitted to maternity and obstetric wards did encounter some level of disrespectful healthcare treatment. All patients should be treated as distinct individuals with respect and dignity, free from discrimination, in accordance with the Maternity Guidelines in South Africa (2023), which are backed by the Patients’ Rights Charter and the Batho Pele principles. For this reason, the term ‘Respectful Maternity Care’ was chosen (Wachira 2019).

As a result, the participants in the study emphasised their favourable experiences with Ubuntu’s midwifery services. Regardless of their age, culture or ethnicity, patients were treated as distinct individuals free from prejudice and discrimination. This finding is reflected by Mulaudzi et al. (2022), who found that leadership from senior midwives is critical in ensuring that student midwives emulate the provision of respectful maternal services.

According to the 2023 Guideline for Maternity Care in South Africa cited in Vedam et al. (2019), midwives are expected to treat women with dignity, compassion and respect regardless of their unfavourable working conditions or any alleged dangerous behaviour during labour. The results of the investigation also detail the disdain that senior midwives showed to the student midwives, who were yelled at while tending to patients. The general lack of regard for others was demonstrated by shouting at patient and student midwives. This was in line with the findings of Manganyi’s (2020) study, which found that poor socialisation during upbringing is the primary cause of this lack of respect.

According to a study by Manganyi (2020), senior midwives and student midwives themselves used harsh, unpleasant language that was unethical and unprofessional, which further highlighted the unpleasant experience caused by yelling at patients. The results are corroborated by Hajizadeh et al. (2020), who found that senior midwives view verbal abuse and disdain towards women as commonplace in an effort to save the mother’s and the unborn child’s lives. Furthermore, according to top midwives, this kind of attitude is required when women refuse to cooperate or follow medical instructions (Hajizadeh et al. 2020).

The more senior midwives’ disregard for the student midwives exemplifies how the respect principle outlined by Jecker, Atuire and Kenworthy (2022) is not being embodied, which has a detrimental effect on the togetherness concept that fosters teamwork. Additionally, the lack of respect among team members is similar to research findings by Jeffery and Wojtalik (2019) who linked the lack of respect and collegiality to high levels of stress and tension between senior midwives and doctors, as well as between senior nurses and student midwives (Jeffery & Wojtalik 2019). The same views are supported by Simane-Netshiasaulu (2021), where participants said that shame, demotivation and frustration caused by senior midwives’ disrespectful remarks and a lack of regard resulted in subpar performance.

It is undeniable that student midwives have typically worked in settings where the Ubuntu principles are applied in a noticeably subpar manner because of staffing shortages, which causes professional midwives to become burned out and then unjustly yell at their patients. This suggests that a staffing deficit may be the cause of potential wrongdoing and ultimately lead to legal action. The extremely inadequate supervision provided by student midwives further exacerbated the latter circumstance. According to the participants, a significant obstacle in the work environment of student midwives was the lack of personnel, which led to the high incidence of stressors like burnout. On the surface, the professional midwives’ yelling at patients might be seen as simple unprofessional behaviour that is attributed to patients who are resistant or disobedient.

Nonetheless, given the current research, this behaviour may be indicative of more severe work-related stress brought on by the workload resulting from the hospitals’ unbalanced nurse–patient ratios. Mathibe-Neke and Masitenyane (2018) have validated these imbalances and their effects, going on to explain that factors including job overload and uneven nurse–patient ratios were likely to contribute to low morale and high staff turnover.

The study’s conclusions regarding the emotional and psychological abuse of patients support those of Maphumulo and Bhengu (2019), who discovered that senior midwives emotionally abuse patients, failing to apply the Ubuntu philosophy of respect, empathy and sympathy to treat them with dignity and respect while they are in the labour ward. Malatjie (2020), who verified that women frequently experienced verbal abuse from midwives during labour, supports the same unfavourable experience. When women don’t comprehend what the nurses demand of them, they are yelled at and rudely spoken to. Because of linguistic and interpretive challenges, the midwives frequently failed to convey what the ladies needed to accomplish. This was especially true for refugees and foreigners.

The situation for both student and senior midwives in maternal service delivery has been made worse by inadequate infrastructure issues in addition to manpower shortages. The successful provision of midwifery care has been identified as being seriously hampered by overcrowding, a lack of beds and a lack of suitable machinery or equipment. One may argue that these issues also reflect shortcomings in the macrocosmic realm of the healthcare system, even though they appear inwardly in the hospitals (Molefe 2019). Such a claim is predicated on the idea that hospitals are unable to address the issue of ward congestion on their own, as the Department of Health has the authority to do so by expanding the current hospitals or constructing new ones.

According to Poorchangizi et al. (2019) regulations such as the *Public Finance Management Act (No. 1 of 1999)*, national or provincial health agencies are responsible for the acquisition and financial management of malfunctioning machinery and equipment. The quotes encapsulated the importance of Ubuntu by stating that the provision of sufficient and skilled human resources guaranteed high-quality maternal services that adhere to Ubuntu. Tolsma et al. (2021) emphasised a similar conclusion, finding that the implementation of Ubuntu was undermined in public institutions of care by models of care that were limited in terms of people and material resources. However, research showed that a pressing problem was also the lack of human resources (Mulaudzi et al. 2022).

Younger professional nurses are working in a resource-poor workplace, according to the participants. The participants emphasised how the delivery of high-quality care is impacted by insufficient funding. The participants further added that because of a staffing shortage, recently trained midwives who were still enrolled in a community service programme as required by SANC were observed helping patients in labour unsupervised. However, Dippenaar (2021) and Jewkes and Penn-Kekana (2019) pointed out that in order to minimise challenges, ensure high-quality care and promote the Ubuntu principles, it is essential to recruit enough midwives who are skilled and competent.

The significance of quality care that embodies Ubuntu and dignified care was summed up in the participant narrative statement. Dehghani et al. (2019) discuss the significance of environmental factors such as infrastructure that facilitate Ubuntu, noting that people’s Ubuntu values are influenced by their surroundings, culture, family, religion and ethnicity. Shunmugam (2022) corroborated the same findings, confirming that inadequate infrastructure in labour wards resulted in a loss of privacy and dignity. The beds, which were near to one another, were crowded. Although nurses utilised curtains to provide seclusion, women may occasionally see and hear what was going on behind them (Shunmugam 2022).

## Strengths and limitations

The study’s strength is its original research strategy, which made it impossible to build it with data that had already been published. Themes were objectively extracted from the collected data and compared to previous findings from the reviewed literature. The interviews were conducted in English, the native language of the individuals. This is beneficial because it gave participants the opportunity to fully express themselves. This implies that the researcher paid attention to what was said and how it was spoken, as well as nonverbal cues, while interpreting the data. As a result, the participants internalised the subliminal messages that were evident in their statements.

The potential constraints of a study do not always indicate a lack of strength. Instead, these limits identify the disciplinary, methodological or practice-related areas that, if overlooked by the researcher, could potentially make the study unsuccessful (Babbie 2020). In that sense, the study’s methodological domain is mostly dominated by potential restrictions. Since only fourth-year midwifery students from the Thohoyandou Nursing Campus and the Department of Advanced Nursing Science, University of Venda were chosen by the researcher, the study may not be generalisable because of its limited sample size. Furthermore, the study’s representative voices may be limited if nurse educators from the Thohoyandou Nursing Campus and the Department of Advanced Nursing Science, University of Venda are not included as participants. Furthermore, the absence of managers or supervisors for the student midwives in the maternity and clinical units may have the same effect.

## Recommendations

Based on the findings, the proposed recommendations relate to the Department of Health, the practice of the midwifery profession, as well as recommendations for future or further research.

### Recommendation for improvement of midwifery service delivery

The results of the study suggest that midwifery practice should incorporate the use of Ubuntu ideals in service delivery. All service areas, including prenatal, intrapartum and postnatal care, should be deeply ingrained with this Ubuntu application. Using Ubuntu in service delivery would improve unethical behaviour that was prevalent in some service units, such as the labour ward, such as yelling at patients, discriminatory practices and disrespect.

### Recommendations for nursing education

Additionally, the study suggests teaching ethical concepts to student midwives while including Ubuntu values. The curriculum’s incorporation of Ubuntu values would encourage the growth of positive attitudes, abilities and application knowledge. Qualified midwives should also be taught Ubuntu as part of ethics through training and seminars.

### Recommendations for further research

The study described student midwives’ experiences applying Ubuntu in Vhembe District using a qualitative methodology. It is advised that future studies employ a quantitative technique that allows the generalisation of study’s findings, given the small sample sizes that are typical of qualitative research.

### Recommendations for the department of health

To guarantee that patients and midwives are aware of service-level requirements, one of the study’s conclusions suggests that the Department of Health post prominent signage on Ubuntu principles in all maternity wards.

### Recommendations for professional midwives

Professional midwives and those tasked with midwifery services in each unit are advised to uphold the Ubuntu values of humanity, respect, equality, cooperation, and compassion. The study also suggests that matrons should make Ubuntu principles a major component of their weekly supervision of certified professional midwives.

## Conclusion

The main goal of the study was to investigate how student midwives at the public hospitals of Vhembe District, Limpopo province, understood and applied the Ubuntu ideals. Such an endeavour is crucial from the standpoint of the researcher because it offers a genuine insight from the very people whose experiences form the basis of a deeper comprehension of the dynamics of midwifery in a genuine and naturalistic setting. The overall results of this study provide a useful reference point for future research by other scholars in the fields of nursing ethics, midwifery and Ubuntu principles, despite the potential restrictions on generalisability mentioned. According to this study, a core goal of midwifery and the provision of healthcare services is the importance of patient care.

The prevalence of certain unprofessional practices and infrastructure issues, as well as the negative consequences they can have in certain situations, did not seem to stop student midwives from learning and putting the Ubuntu values of subordination, caring and togetherness and/or teamwork into practice as part of their core responsibilities. Furthermore, the researchers argue that the future of Ubuntu ideals is not in jeopardy because the majority of nurses and midwives are younger than their older, more seasoned and senior peers. Accordingly, it is determined that midwifery’s vitality can be sustained, so long as a corresponding change is implemented and formalised in South Africa’s nursing education and training framework.
